# The Effect on Fertility of the 2003–2011 War in Iraq

**DOI:** 10.1111/j.1728-4457.2014.00001.x

**Published:** 2014-12-12

**Authors:** Valeria Cetorelli

**Affiliations:** London School of Economics and Political Science

## Abstract

This article provides the first detailed account of recent fertility trends in Iraq, with a particular focus on the changes resulting from the 2003–2011 war and the factors underlying them. The study is based on retrospective birth history data from the 2006 and 2011 Iraq Multiple Indicator Cluster Surveys (I-MICS). Estimates from the two surveys indicate that total fertility remained stable from 1997 to 2010, at about 4.5 children per woman. However, examination of the age patterns of fertility reveals an abrupt shift in the timing of births, with adolescent fertility rising by over 30 percent soon after the onset of the war. A decomposition analysis shows that the rise in early childbearing is due to an increased prevalence of early marriage among less-educated women. The prevalence of early marriage and childbearing among women with secondary or higher education is relatively low and has not increased after 2003.

The increasing concern about the consequences of warfare for civilian populations has led to a growing body of demographic research. This research has been essential in providing estimates of war-induced excess mortality, a primary indicator by which to assess the intensity of wars and the adequacy of humanitarian responses.[1] Research on war-induced changes in fertility is much scarcer, although the need to monitor women's status and reproductive health in war zones has been widely acknowledged (Palmer, Lush, and Zwi 1999; McGinn 2000; McGinn and Purdin 2004; Austin et al. 2008; McGinn 2009; Patel et al. 2009). This is especially evident in the case of the 2003–2011 war in Iraq. Several studies have sought to quantify the impact of this war on mortality (e.g., Roberts et al. 2004; Burnham et al. 2006; Iraq Family Health Survey Study Group 2008; Hagopian et al. 2013). By contrast, virtually nothing is known about the effect of this war on fertility.

This article provides the first detailed account of recent fertility trends in Iraq, with a particular focus on the changes resulting from the war and the factors underlying them. The analysis is based on retrospective birth history data from the 2006 and 2011 Iraq Multiple Indicator Cluster Surveys (I-MICS). To assess the quality of the data, I calculate retrospective fertility rates from each survey by single calendar year and compare fertility estimates from one survey with the estimates for the same period from the subsequent survey. I then pool the data to reconstruct annual fertility trends from 1997 to 2010, allowing for comparisons over a period spanning before and after the onset of the war. Using decomposition techniques, I quantify how much of the change in fertility rates during wartime was accounted for by changes in the proportion of married women and how much by changes in the prevalence of birth control within marriage. The findings have implications for the design of women's empowerment and health strategies in Iraq and similar war-affected settings.

## Background

The number of studies assessing fertility changes during war in low- and middle-income countries is scant (Hill 2004; Guha-Sapir and D'Aoust 2010).[2] The limited evidence suggests that these changes vary not only in magnitude but also in direction, depending on the nature of the war and the pre-existing characteristics of the affected population.

War-induced fertility declines have been documented in a few sub-Saharan African countries during the 1980s and 1990s. These declines have been attributed to involuntary spousal separation and postponement of new marriages attributable to military mobilization, accompanied in some cases by voluntary efforts to delay or avoid childbearing as a response to economic hardship.[3] War-induced fertility increases have also been observed in some Middle Eastern countries during the same decades. These increases have been accounted for by a rise in the proportion of young women married in response to security concerns and lack of alternatives, and from limited birth control within marriage owing to war-induced pronatalist ideologies and disruption of family planning services.[4]

To date no study has assessed changes in fertility that may have resulted from more recent wars affecting the Middle East since the early 2000s, including the 2003–2011 Iraq War.[5] Fertility trends in Iraq during this war, as well as during the preceding period, have remained largely undocumented (Tabutin and Schoumaker 2005; Casterline 2009). It is likely that these trends have been affected by the country's turbulent history and associated population policies.

Saddam Hussein's regime embraced a strong pronatalist ideology since its inception in 1979. High fertility was encouraged through various economic incentives, including childbirth cash bonuses and family allowances (Faour 1989). The 1981–1988 war against Iran and the Iranian superiority in population size led Saddam Hussein to further intensify the country's pronatalist approach, with the aim of accelerating population growth. Major restrictions on access to contraception were put in place, and penalties for performing illegal abortions were increased. Family planning services provided by the Iraqi Family Planning Association and the private sector were reserved exclusively for medical reasons (United Nations 1987; Efrati 1999). Only in the aftermath of the 1990–1991 Gulf War did the regime finally issue a decree allowing the provision of family planning services to all women. During the 1990s and early 2000s, the Iraqi Family Planning Association received international assistance under a special dispensation from the UN embargo, but a contraceptive shortage persisted (United Nations 2001).[6]

The conditions prevailing during the 2003–2011 Iraq War are likely to have altered previous fertility trends in multiple, and possibly countervailing, ways. On the one hand, the regime change marked a turning point in the country's population policy. In the post-2003 period, family planning services have been actively supported by the government (United Nations 2011). The wider availability of birth control methods may have fostered a decline in the average number of children per woman. On the other hand, the post-2003 period has witnessed a deterioration in women's status as a result of widespread violence and rising conservatism (Brown and Romano 2006; UNICEF 2011). The dire security situation, combined with the resurgence of sectarian, tribal, and generally conservative forces, has severely restricted women's possible roles outside the home and may have induced many to enter marriage and childbearing earlier than they might have in the absence of war.

The following analysis reveals how fertility changed during wartime and identifies the factors underlying these changes. The analysis excludes the autonomous Iraqi Kurdistan region, which experienced very different conditions. This region consists of the three northern governorates of Dohuk, Erbil, and Al-Sulaimaniya, accounting for about 15 percent of the Iraqi population. After the 1991 Kurdish uprising against Saddam Hussein's regime, these governorates became a de facto autonomous region under UN auspices. As a result, during the sanction period family planning methods were more easily available than elsewhere in the country. Unlike the rest of Iraq, this region witnessed virtually no combat during the 2003 US-led invasion and has not suffered from generalized violence and insecurity in the subsequent years. (A supplemental appendix, available at wileyonlinelibrary.com/journal/padr, shows that fertility trends in Kurdistan were not altered after 2003.)

## Data

This study is based on retrospective birth history data from the 2006 and 2011 I-MICS. These nationally representative surveys were conducted by the Iraqi Central Organisation for Statistics and Information Technology and the Kurdistan Regional Statistics Office, in coordination with the Ministry of Health and with financial and technical support from UNICEF (Iraq Central Organisation for Statistics and Information Technology and Kurdistan Regional Statistics Office 2007, 2013). In this section, I describe survey designs, implementation, and response rates, excluding the sample from the autonomous Iraqi Kurdistan region.

For the 2006 I-MICS, the country was divided into 47 domains, and 54 clusters were drawn from each domain with linear systematic probability proportional to size. A linear systematic sample of six households was selected within each cluster. Fieldwork took place between February and March 2006, and the household questionnaire was administered to 15,085 of the selected households, yielding a response rate of 99.2 percent (Iraq Central Organisation for Statistics and Information Technology and Kurdistan Regional Statistics Office 2007). For the 2011 I-MICS, 31 clusters were selected within each of the 85 Iraqi districts with linear systematic probability proportional to size, and ten households were drawn from each cluster by systematic random sampling. Fieldwork was carried out between March and May 2011, reaching 25,984 households for a response rate of 99.7 percent (Iraq Central Organisation for Statistics and Information Technology and Kurdistan Regional Statistics Office 2013).

In both surveys, the household questionnaire was administered to the head of each household for, among other purposes, gathering information about the age and sex of all household members. All women between ages 15 and 49 who were members of the selected households were eligible for the individual interview. Women's response rate was 99.1 percent in the 2006 I-MICS and 98.7 percent in the 2011 I-MICS. Retrospective birth histories were collected for all interviewed women who were ever-married at the date of the interview.

Complete and accurate information regarding the birth date of women and the date of each of their live births is crucial for a correct estimation of fertility trends. In countries, like Iraq, with flawed vital registration systems, surveys collecting birth histories from a nationally representative sample of women are the most reliable sources for this purpose. Nevertheless, reporting errors are critical problems for many of these surveys (Potter 1977; Goldman, Rutstein, and Singh 1985; Arnold 1990; Marckwardt and Rutstein 1996; Pullum 2006; Schoumaker 2011; Pullum et al. 2013). Since these errors can distort fertility estimates, it is important to determine the quality of the data.

Both the 2006 and 2011 I-MICS are of good quality in terms of birth date completeness. Information regarding month and year of birth is complete for 95.6 percent of interviewed women and 97.2 percent of their reported live births in the 2006 I-MICS, and for 99.6 percent of women and 99.5 percent of reported live births in the 2011 I-MICS. However, complete reporting does not necessarily imply accurate reporting.

The two most widely mentioned and potentially serious problems with birth history data are omission and displacement of births (Pullum 2006; Sullivan et al. 2008; Schoumaker 2011; Pullum et al. 2013). A common error is the omission of births that occurred many years before the survey, especially in the case of deceased children (Sullivan 2008). Omission and displacement of recent births are also common and related to the design of the survey questionnaires. Like many similar surveys, the 2006 and 2011 I-MICS contained a child health module only for those women with children born up to five years before the survey. Previous research has found that interviewers are inclined to omit some recent births to avoid administering the lengthy health module (Schoumaker 2011). The same reason encourages interviewers to displace some recent births backward in time, particularly from the fifth year before the survey, which is the last year of eligibility for the health module, to the sixth year (Pullum 2006). These tactics tend to cause an underestimation of fertility in the five years preceding the surveys and an overestimation in the sixth year.

When two consecutive surveys are available, as they are here, an effective approach to detect omission and displacement of births and assess the overall reliability of the birth history data is to reconstruct retrospective fertility trends from each survey and compare fertility estimates from one survey with the estimates for the same period from the subsequent survey. In the absence of distortions resulting from data errors, I expect the 95 percent confidence intervals of the estimated fertility rates from the 2006 and 2011 I-MICS to overlap.[7]

## Reconstructing fertility trends

Birth history data from the 2006 and 2011 I-MICS can be used to reconstruct annual fertility rates from 1997 to 2010. This makes it possible to depict trends for a period spanning before and after the onset of the war. The fertility rate of women aged $x$ in calendar year $t$ is:

$$f_{x,t} = b_{x,t} / e_{x,t} \tag{1}$$

where $b_{x,t}$ is the number of births observed in calendar year $t$ to women aged $x$ at the time of birth, and $e_{x,t}$ is the number of women-years of exposure to risk at age $x$ during year $t$. The 95 percent confidence intervals for $f_{x,t}$ are derived from standard errors computed using the delta method (Pullum 2008; Schoumaker 2013). Because of truncation of the data on older women, the analysis of retrospective fertility rates is restricted to women aged 15–39. Since very few births occur to Iraqi women at age 40 and older, the downward bias of fertility due to the omission of this small fraction of births is negligible. Finally, it is important to state that these estimates are based on a sample of surviving women residing in Iraq at the time of the two surveys. Women who died or left the country during the war are thus excluded.

Figure 1a displays trends in the total fertility rate (TFR) for women aged 15–39 as estimated from the two surveys. Estimates from the 2011 I-MICS cover the period 2002–2010. The reason is that the oldest women whose birth histories were collected in the 2011 I-MICS were aged 49 at the date of the interview, and were therefore aged 39 ten years earlier. Birth histories from the 2006 I-MICS cover the period 1997–2005. Estimates from the two surveys agree closely with each other. Only for the year 2005 do the 95 percent confidence intervals not overlap. The difference between the two TFRs in 2005 may result from both a slight underestimation of fertility in the year before the 2006 I-MICS, due to omission of births, and an

**FIGURE 1 TFRs for women aged 15–39, Iraq 1997–2010**

overestimation of fertility in the sixth year before the 2011 I-MICS, due to displacement of births from the earlier period. Although the estimated TFR for 2005 must be treated with caution, the difference between the two rates is not large enough to distort the overall fertility trend, which is consistent between the two surveys.

Figure 1b shows the estimated trend in the TFR using pooled data from the 2006 and 2011 I-MICS. Pooled fertility rates are obtained by summing the number of births from the two surveys in year $t$ to women aged $x$ at time of birth and dividing them by the sum of the number of women-years of exposure. Two noteworthy points emerge. First, total fertility in 2010 was still around 4.5 children per woman, which is fairly high compared to Iraq's neighboring countries.[8] Second, the TFR remained remarkably stable, with apparently no change after the onset of the war. Total fertility in 2010 was exactly the same as in 1997, and annual fluctuations remained below 10 percent over the entire period.

However, the TFR reflects both the level and timing of fertility, and its stability over time can conceal underlying changes in the age patterns of childbearing. To uncover possible variations in fertility trends among women of different age groups, Figure 2a displays age-specific fertility rates (ASFRs) from the 2006 and 2011 I-MICS. Birth histories from the 2011 I-MICS can be used to calculate retrospective fertility rates back to 1997 for the age groups 15–19, 20–24, 25–29, and 30–34. This means that, for these groups of women, the estimated ASFRs from the two surveys can be compared for nine years. Over this longer time period and at this less aggregate level, fertility trends are consistent. The 95 percent confidence intervals of fertility estimates fail to overlap in 2005 only for the age groups 20–24 and 30–34. For all other age groups of women, and in particular for those aged 15–19, estimates from the two surveys agree remarkably well for all nine years.

Figure 2b presents the estimated ASFRs pooling data from the two surveys. This figure reveals that the stability of the TFR before and after the onset of the war was the result of countervailing fertility trends among younger and older women. To examine these trends in more detail, Figure 3 displays the annual changes in ASFRs relative to the 1997 rates. The figure shows an abrupt shift in the timing of births toward younger ages. From 1997 to 2003, adolescent fertility was stable at just below 70 births per 1,000 girls aged 15–19. However, soon after the beginning of the war, adolescent fertility rose by more than 30 percent, reaching over 95 births per 1,000 girls in 2010. This increase is striking not only because it moved Iraq from moderate to high adolescent fertility,[9] but also because a similar increase in adolescent fertility over such a short period has rarely been observed.[10]

The fertility rate of women aged 20–24 also increased after the onset of the war by about 15 percent, from 200 to 230 births per 1,000 women in that age group. Only the fertility of women aged 25–29 remained virtually

**FIGURE 2 ASFRs for women aged 15–39, Iraq 1997–2010**

**FIGURE 3 ASFRs for women aged 15–39 relative to 1997 rates: Iraq, 1997–2010 (Index: 1997 = 100)**

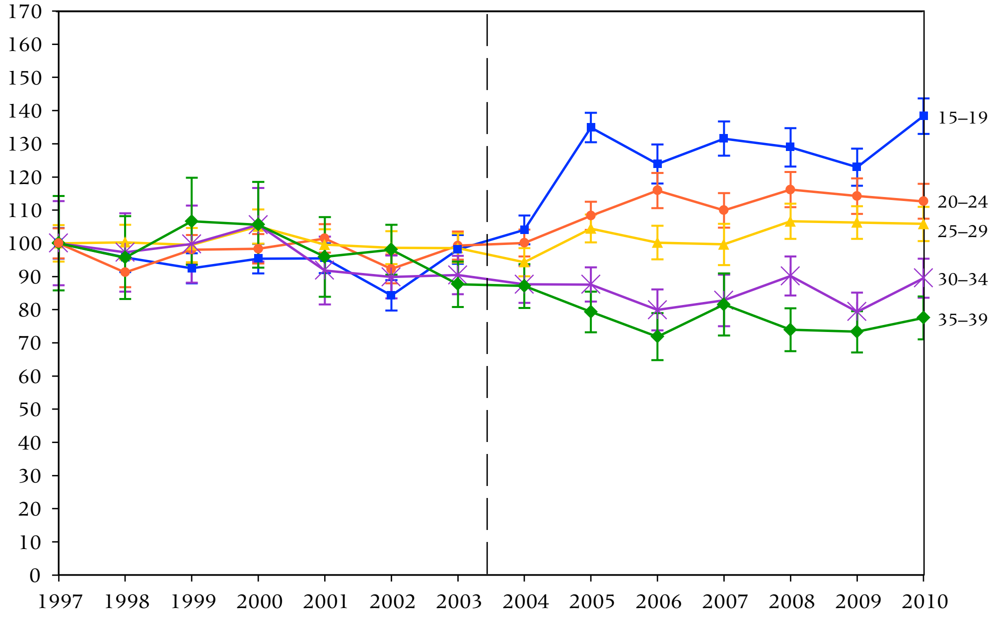

SOURCE: Pooled data from 2006 and 2011 I-MICS.

unchanged at around 250 births per 1,000. The increase in early fertility was counterbalanced by a 15 percent decline in the fertility of women aged 30–34, from 230 to 200 births per 1,000, and a 30 percent decline among those aged 35–39, from 170 to 130 births per 1,000. It is noteworthy that the fertility rates of young women rose suddenly in the post-2003 period, whereas the declining fertility trends among older women were already underway a few years before the beginning of the war.

## Distinguishing fertility trends by education

The abrupt rise in early fertility is most likely related to an increase in early marriage during the war. Virtually all births in Iraq occur within marriage, since extramarital childbearing is subject to strong cultural and religious sanctions. Once married, women are generally under social pressure to have children as soon as possible. Data from the 2006 and 2011 I-MICS confirm that only 1 percent of married women use contraception before having at least one child.[11]

Previous research has found that in low- and middle-income countries women's education is the most important predictor of age at marriage and first birth. Having secondary education specifically is the factor most strongly associated with reduced prevalence of adolescent marriage and childbearing (Jain and Kurz 2007; Myers and Harvey 2011). In what follows, I examine fertility differentials between women with no education or only primary schooling and women who attended (but did not necessarily complete) secondary or higher education. The aim is to determine whether there was a change in the age pattern of childbearing in one or both educational groups. According to the 2006 and 2011 I-MICS, the proportion of women with less than secondary education is around 55 percent, with little variation across age groups.

Figure 4a shows annual trends in the education-specific TFR as estimated from the 2006 and 2011 I-MICS. Estimates are consistent between the two surveys, with overlapping 95 percent confidence intervals except in 2005 for less-educated women. Trends using pooled data are reported in Figure 4b. This figure reveals a significant educational gap in TFR, with less-educated women having on average 1.5 more children than highly educated women. The gap in total fertility between the two groups remained relatively constant over the entire period.

Once again this overall stability conceals very different trends in the age patterns of fertility between women with low and high education. Figure 5a presents ASFRs by survey for less-educated women. Estimates from the two surveys agree closely with each other. The 95 percent confidence intervals of these estimates fail to overlap in 2005 only for women aged 30–34. Figure 5b shows estimates using pooled data, and Figure 6 uses these estimates to display the annual changes in ASFRs relative to the 1997 rates. The shift in the timing of fertility toward younger ages was much more pronounced among women with less than secondary education than it appeared at the aggregate level.

Soon after the onset of the war, adolescent fertility rose by over 50 percent, from about 85 to 135 births per 1,000 girls. Fertility increased by over 15 percent among women aged 20–24, from 230 to 270 births per 1,000, and remained stable among women aged 25–29 at around 270 births per 1,000. The fertility decline at older ages was also more pronounced among less-educated women than at the aggregate level. Fertility dropped by 20 percent among women aged 30–34, from 260 to 220 births per 1,000, and by 40 percent among those aged 35–39, from 190 to 135 births per 1,000.

The ASFRs for women with secondary or higher education by survey are reported in Figure 7a. Fertility estimates for this group are more erratic because of the smaller number of births. Yet, the 95 percent confidence intervals of these estimates overlap for all age groups over the entire period, except in 2000 and 2005 for women aged 20–24. Figure 7b shows the estimated ASFRs using pooled data, and Figure 8 displays the relative changes in ASFRs compared to the 1997 rates. Before the war, highly educated women exhibited lower fertility than less-educated women in all age groups. During

**FIGURE 4   Education-specific TFRs for women aged 15–39, Iraq 1997–2010**

**a. Data from separate 2006 and 2011 surveys**

**b. Pooled data**

**FIGURE 5 ASFRs for less-educated women aged 15–39, Iraq 1997–2010**

**a. Data from separate 2006 and 2011 surveys**

**b. Pooled data**

**FIGURE 6 ASFRs for less-educated women aged 15–39 relative to 1997 rates: Iraq, 1997–2010 (Index: 1997 = 100)**

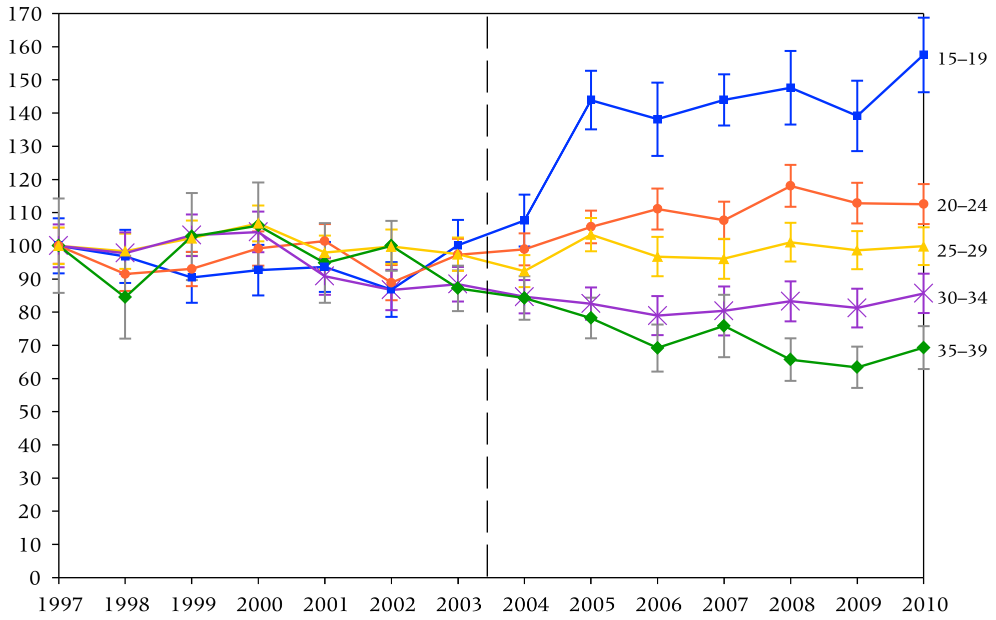

SOURCE: Pooled data from 2006 and 2011 I-MICS.

the war, highly educated women did not experience any significant fertility change. Fertility fluctuated around 45 births per 1,000 among adolescents, 160 births among women aged 20–24, 210 at ages 25–29, 180 at ages 30–34, and 130 at ages 35–39.

The shift toward early childbearing that occurred only among less-educated women led to a widening of fertility differentials by education among the youngest age groups, especially adolescents. In 2010 the contribution of adolescent fertility to the TFR among less-educated women was over 90 percent higher than among highly educated women, whereas in 1997 it was just over 30 percent higher. On the other hand, the educational gap in fertility at older ages gradually narrowed, and in 2010 less-educated women aged 35–39 had the same fertility rate as highly educated women in that age group.

A number of questions arise. What were the factors underlying the observed fertility changes among less-educated women? Was the sudden rise in fertility at younger ages determined by a higher prevalence of early marriage during the war? And what was the role of birth control use on falling fertility at older ages? The remainder of the article seeks to answer these questions.

**FIGURE 7 ASFRs for highly educated women aged 15–39, Iraq 1997–2010**

**a. Data from separate 2006 and 2011 surveys**

**b. Pooled data**

**FIGURE 8 ASFRs for highly educated women aged 15–39 relative to 1997 rates: Iraq, 1997–2010 (Index: 1997 = 100)**

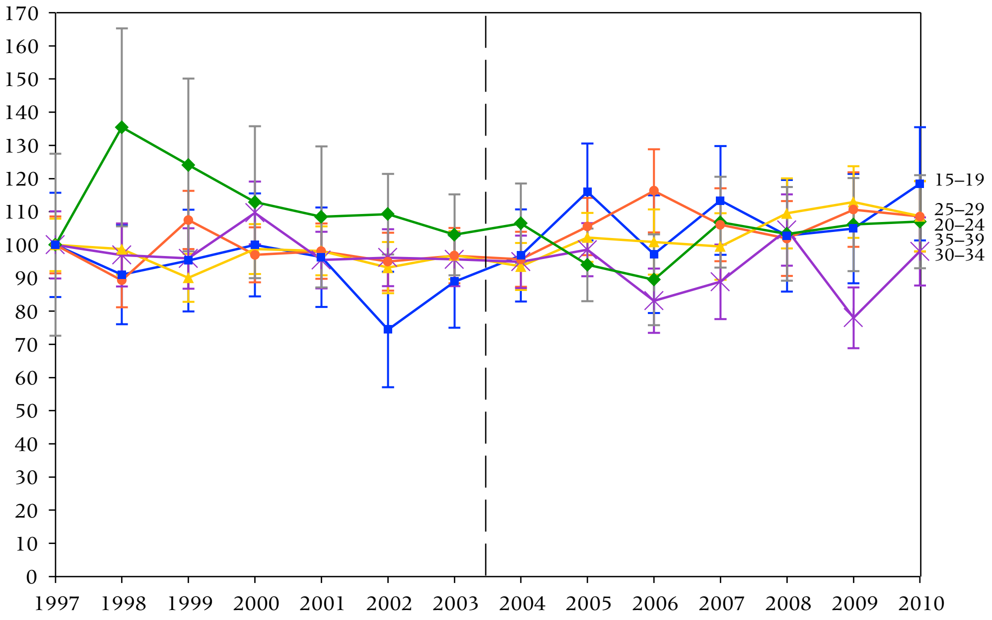

SOURCE: Pooled data from 2006 and 2011 I-MICS.

## Decomposing fertility changes among less-educated women

In this section, I quantify the relative contribution of marital composition and marital fertility to the observed fertility changes among less-educated women before and after the onset of the war. Under the assumption that all births occur within marriage, the fertility rate of women aged *x* in calendar year *t* can be rewritten as:

$$f_{x,t} = \left(\frac{b_{x,t}}{e_{m,x,t}}\right)\left(\frac{e_{m,x,t}}{e_{s,x,t} + e_{m,x,t}}\right) \tag{2}$$

where $e_{m,x,t}$ are the number of women-years of exposure at age *x* during calendar year *t* that are spent married, and $e_{s,x,t}$ are the number of women-years that are spent single, the sum of the two being equal to the total number of women-years $e_{x,t}$. In other words, the fertility rate of women at a given age in a given year is the product of the age-year-specific marital fertility rate $f_{m,x,t}$ and the age-year-specific proportion of women-years of exposure spent within marriage $p_{m,x,t}$ :

$$f_{x,t} = f_{m,x,t} * p_{m,x,t} \tag{3}$$

Accordingly, the change in the fertility rate from year $t$ to year $t{+}n$ can be decomposed as:

$$f_{x,t+n} - f_{x,t} = \begin{array}{l} [\tfrac{1}{2}(f_{m,x,t+n} + f_{m,x,t}) * (p_{m,x,t+n} - p_{m,x,t})] \\ + [(f_{m,x,t+n} - f_{m,x,t}) * \tfrac{1}{2}(p_{m,x,t+n} + p_{m,x,t})] \end{array} \qquad (4)$$

where the first of the two main components on the right side of equation 4 gives the proportion of the change in the fertility rate stemming from change in marital composition, and the second component gives the proportion stemming from change in marital fertility.[12] I use equation 4 to decompose the observed fertility changes among less-educated women comparing the pre- and post-2003 period.

Table 1 displays age-specific percentages ever-married and marital fertility rates for less-educated women in 1997, 2003, and 2010. Estimates are based on pooled data from the 2006 and 2011 I-MICS. The use of ever-married rather than currently married women in a specific calendar year is dictated by data availability. The 2006 and 2011 I-MICS collected information on the age at first marriage for all ever-married women, but did not ask about the duration of marriage for those who were divorced or widowed at the date of the interview.

From 1997 to 2003, marital exposure to fertility remained virtually identical for adolescents and young adult women. The percentages ever-married were around 21 percent among those aged 15–19 in both years, and 57 percent in 1997 and 56 percent in 2003 for those aged 20–24. Among older age groups, the percentages ever-married declined slightly during this period, from around 79 percent to 76 percent among women aged 25–29, from 90 percent to 84 percent among those aged 30–34, and from 94 percent to 92 percent among those aged 35–39.

Meanwhile, marital fertility remained relatively stable among the youngest age groups, whereas it exhibited a clear declining trend at older ages. The fertility of married women aged 30–34 dropped from about 291 births per 1,000 in 1997 to 273 in 2003. The decline was more pronounced

**TABLE 1 Marital composition and marital fertility among less-educated women: Iraq, 1997, 2003, and 2010**

| | Percent ever-married | | | Marital fertility rate | | |
|---|---|---|---|---|---|---|
| **Age group** | **1997** | **2003** | **2010** | **1997** | **2003** | **2010** |
| 15–19 | 21.4 | 21.4 | 32.0 | 402.8 | 403.5 | 423.3 |
| 20–24 | 57.2 | 56.1 | 70.3 | 421.1 | 417.7 | 385.9 |
| 25–29 | 78.8 | 76.0 | 84.8 | 344.3 | 347.9 | 319.3 |
| 30–34 | 89.5 | 84.4 | 87.9 | 290.5 | 272.6 | 253.7 |
| 35–39 | 93.8 | 92.1 | 89.9 | 207.5 | 184.2 | 150.1 |

SOURCE: Pooled data from 2006 and 2011 I-MICS.

among those aged 35–39, from 208 to 184 births per 1,000. The fact that in 2003 married women were stopping childbearing earlier than they did in 1997 suggests that the fertility transition was underway, despite the prolonged pronatalist ideology of Saddam Hussein's regime and the contraceptive shortage in the country. Given the deterioration of living conditions during the embargo in the 1990s and early 2000s, this decline in the marital fertility of less-educated women may have been the result of economic hardship (Baram 2000).

The period 2003–2010 saw a sharp rise in marital exposure among the youngest age groups, with little change at older ages. The proportions ever-married increased from about 21 percent to 32 percent among adolescents, from 56 percent to 70 percent among women aged 20–24, and from 76 percent to 85 percent among those aged 25–29. The fact that the proportion ever-married at ages 35–39 remained relatively stable at around 90 percent indicates that during the war women who would instead have married later in life entered marriage at much younger ages. As mentioned at the beginning of this article, women's activities and possible roles outside the home were severely restricted after 2003 by security concerns and increased sectarian and religious conservatism (Brown and Romano 2006; UNICEF 2011). The wider prevalence of early marriage compared to the pre-2003 period may therefore be essentially due to a lack of alternatives for less-educated adolescents and young adult women.

The increase in marriage during the war was accompanied by a substantial drop in marital fertility across all age groups other than adolescents. This suggests that a larger number of married women were using contraception both to space births and to stop childbearing. The increased use of birth control within marriage was certainly facilitated by the wider availability of family planning information and services following the regime change in 2003. Nevertheless, it is likely that the reasons for falling marital fertility among less-educated women were still largely related to economic hardship. It is also possible that a fraction of this decline was the result of an increase in widowhood, divorce, and spousal separation during the war.

Results of the fertility decomposition are provided in Table 2. The first column summarizes fertility changes among less-educated women during the period 1997–2003. Total fertility declined only slightly during this period from 5.3 children per woman in 1997 to 4.9 in 2003. Almost 90 percent of this decline was accounted for by lower fertility among women aged 30–34 and 35–39. Fertility remained virtually unchanged at younger ages, especially among adolescents. The second and third columns show that about half of the fertility decline among women aged 30–34 was accounted for by a reduction in proportions married and about half by a reduction in marital fertility. For the age group 35–39, over 80 percent of the decline was attributable to reduced marital fertility.

**TABLE 2 Decomposition of changes in the fertility rates of less-educated women into changes related to marital composition and to marital fertility: Iraq, 1997–2003 and 2003–2010**

| | 1997–2003 | | | 2003–2010 | | |
|---|---|---|---|---|---|---|
| Age group | Total change | Marital composition | Marital fertility | Total change | Marital composition | Marital fertility |
| 15–19 | 0.00 | 0.00 | 0.00 | 0.25 | 0.22 | 0.03 |
| 20–24 | –0.03 | –0.02 | –0.01 | 0.18 | 0.28 | –0.10 |
| 25–29 | –0.04 | –0.05 | 0.01 | 0.03 | 0.14 | –0.11 |
| 30–34 | –0.15 | –0.07 | –0.08 | –0.03 | 0.05 | –0.08 |
| 35–39 | –0.13 | –0.02 | –0.11 | –0.18 | –0.02 | –0.16 |
| Total | –0.35 | –0.16 | –0.19 | 0.25 | 0.67 | –0.42 |

SOURCE: Pooled data from 2006 and 2011 I-MICS.

During the period 2003–2010, total fertility among less-educated women rose from 4.9 to 5.2 children per woman. This change was the result of two countervailing fertility trends: a fertility increase at younger ages and a continuation of fertility decline at older ages. Fertility in the age group 15–19 increased by 0.25 children per woman, with about 90 percent of the increase resulting from a wider prevalence of adolescent marriage. In the age group 20–24 fertility increased by 0.18 children per woman. This increase was over-determined by a wider prevalence of marriage. Other things being equal, the rise in marriage among these women would have increased fertility by 0.28 children. However, the rise in proportions married was partially offset by a reduction in marital fertility. The relative stability of fertility among women aged 25–29 was also the result of a combination of increased marital exposure and reduced marital fertility. On the other hand, the fertility decline in the age group 30–34 was over-determined by falling marital fertility, and the decline in the age group 35–39 was accounted for almost completely by falling marital fertility.

## Conclusion

This study is the first detailed account of fertility changes in Iraq during the 2003–2011 war. I have shown that the apparent stability of the TFR at 4.5 children per woman was the result of two countervailing trends in age at marriage and marital fertility. On the one hand, the decline in marital fertility, which was already underway before 2003, accelerated during the war. The decline was mostly concentrated among less-educated women and was likely to have been poverty-driven. Living conditions in Iraq deteriorated during the embargo and have failed to improve after 2003. Nearly 25 percent of Iraqis live below the poverty line, and many families still rely on the public distribution system for basic food items (United Nations 2014; Rawaf et al.

2014). Birth control within marriage may have been fostered by the wider availability of family planning services following the regime change and the end of the embargo.

On the other hand, the post-2003 period has witnessed an abrupt shift in the timing of fertility toward younger ages. Adolescent fertility in particular increased sharply after the onset of the war. This is the result of an increased prevalence of early marriage, most likely as a response to the dire security situation and rising conservatism throughout the country. Insecurity, especially the actual and perceived dangers of harassment, combined with the resurgence of sectarian, tribal, and other conservative forces, prevent many women from participating in public life or even from leaving their homes without the escort of a male relative. In this context young women may be induced to marry early by a lack of alternatives, while families may consider early marriage as the best way to protect their daughters and the family's honor. The prevalence of early marriage may have further increased after 2011, the result of a new escalation of insurgency and sectarian violence following the departure of the last US armed forces.

This trend is worrisome because it is widely acknowledged that women who marry during adolescence tend to have lower status in the home and that adolescents who bear children face higher risks of maternal mortality and morbidity as well as poorer health outcomes for their children. The deleterious effects may be magnified by the fact that this phenomenon is concentrated among women with little education. The prevalence of early marriage and childbearing among women with secondary or higher education is relatively low and has not increased after 2003.

## Notes

This study was supported by a UK Economic and Social Research Council Doctoral Studentship and an Emirates Foundation Award. The author thanks Stephen Jenkins, Tiziana Leone, and Ben Wilson for helpful comments and suggestions.

1 Since the early 2000s, a large number of studies on mortality in war-affected countries have been conducted. See for example Spiegel and Salama (2000) for Kosovo; Grein et al. (2003) for Angola; Tabeau and Bijak (2005) for Bosnia and Herzegovina; Roberts et al. (2001), Roberts et al. (2003), and Coghlan et al. (2006) for the Democratic Republic of Congo; Depoortere et al. (2004), Hagan and Palloni (2006), and Degomme and Guha-Sapir (2010) for Darfur; and Roberts et al. (2004), Burnham et al. (2006), Iraq Family Health Survey Study Group (2008), and Hagopian et al. (2013) for Iraq.

2 Fertility declines in European countries during World Wars I and II are well-known. These declines were largely attributable to spousal separation and the drop in the number of new marriages due to conscription. See among others Vincent (1946), Hajnal (1947), Winter (1992), Festy (1984), and Chesnais (1992).

3 See Lindstrom and Berhanu (1999) for the Ethiopian civil war; Agadjianian and Prata (2002) for the Angolan civil war; and Blanc (2004) and Woldemicael (2008) for Eritrea's border war against Ethiopia.

4 See Khawaja (2000) for the first Palestinian Intifada; Abbasi-Shavazi et al. (2002) for Iran's war against Iraq; and Yüceşahin and Özgür (2008) for the Kurdish guerrilla in southeastern Turkey.

5 The Iraq War began in March 2003 when US-led military forces invaded Iraq and removed Saddam Hussein's regime from power. Since then, the country has experienced insurgent and sectarian violence mostly targeting civilians. While insurgency and hostilities have continued, the departure of the last US forces in December 2011 is conventionally considered to mark the end of the war.

6 Iraq's invasion of Kuwait in August 1990 led to the immediate imposition of a strict economic embargo by the UN Security Council. The embargo was maintained and extended after the Gulf War. The only imports permitted into Iraq were food, medicine, and other items classified as humanitarian aid. An Oil-for-Food Program was approved by the UN Security Council in April 1995, allowing Iraq to sell oil to purchase humanitarian supplies under UN supervision. The embargo was lifted in May 2003.

7 For a similar approach see Garenne (2008), Schoumaker (2009), Machiyama (2010), and Pullum et al. (2013).

8 During the late 2000s, total fertility in Iraq's neighboring countries ranged from about 2 to 3.5 children per woman. See for example Abbasi-Shavazi et al. (2009) for Iran; Adbul Salam (2013) for Saudi Arabia; Al-Kandari (2007) for Kuwait; Cetorelli and Leone (2012) for Jordan; Yavuz (2005) for Turkey; and Youssef (2012) for Syria.

9 According to the United Nations, a country is classified as having a high rate of adolescent fertility if it has over 80 births per 1,000 girls aged 15–19 (United Nations 2013).

10 A substantial increase in adolescent fertility was documented in the Palestinian Territories during the first Intifada (see Khawaja 2000).

11 The age-specific prevalence of modern contraceptive use ranges from 15 percent among ever-married women aged 15–19 to over 45 percent among those aged 35–39.

12 This decomposition method was introduced by Kitagawa (1955) to decompose changes in crude birth rates and was extended to fertility rates by Retherford and Ogawa (1978) and Retherford and Rele (1989). Since then, this method, with various adaptations, has been used extensively to analyze fertility changes over time in diverse populations. See for example Khawaja (2000), Lindstrom and Woubalem (2000), Retherford et al. (2005), Abbasi-Shavazi et al. (2009), and Gubhaju, Jongstra, and Raikoti (2013).
