# Supplementary material for: The Effect on Fertility of the 2003–2011 War in Iraq
Source: Popul Dev Rev. 2014 Dec 12;40(4):581–604. doi: 10.1111/j.1728-4457.2014.00001.x (PMC4539598; doi:10.1111/j.1728-4457.2014.00001.x)
Supplement: Supplementary file 1 — Supporting Information [file padr0040-0581-sd1.pdf]

Fertility trends in the autonomous Iraqi Kurdistan region

The 2006 and 2011 I-MICS collected birth history data in the autonomous Iraqi Kurdistan region as well as in the rest of the country. For the 2006 I-MICS, the region was divided into 9 domains. Like elsewhere, 54 clusters were drawn from each domain with linear systematic probability proportional to size and a linear systematic sample of six households was selected within each cluster. Fieldwork in the region took place between April and June 2006. The household questionnaire was successfully administered to 2,778 of selected households, yielding a response rate of 95.8 percent (Iraq Central Organisation for Statistics and Information Technology and Kurdistan Regional Statistics Office 2007). For the 2011 I-MICS, 31 clusters were selected within the 33 Kurdish districts with linear systematic probability proportional to size and ten households were drawn from each cluster by systematic random sampling. Fieldwork was carried out between February and March 2011, reaching 9,717 households for a response rate of 99.5 percent (Iraq Central Organisation for Statistics and Information Technology and Kurdistan Regional Statistics Office 2013).

Survey data in this region are of similarly good quality as in the rest of Iraq. Women's response rate is 95.7 percent in the 2006 I-MICS and 95.2 percent in the 2011 I-MICS. Information regarding month and year of birth is complete for 97.5 percent of interviewed women and 95.3 percent of their reported live births in the 2006 I-MICS, and for 99.7 percent of women and 97.8 percent of reported live births in the 2011 I-MICS.

Figure A1a shows trends in the TFR for the Kurdistan region as estimated from the 2006 and 2011 I-MICS. The estimates from the two surveys agree closely with each other, with the 95 percent confidence intervals overlapping in each year. Figure A1b displays the estimated trend in the TFR pooling data from the two surveys. Total fertility declined steeply, from an average rate of 5 children per woman in the late 1990s to 3 children per woman in 2010.

The ASFRs by survey are reported in figure A2a. Due to the small sample size, the trends are more erratic and the 95 percent confidence intervals are larger than those presented previously for the rest of Iraq. Nevertheless, estimates from the two surveys are consistent for all age groups over the entire period. Figure A2b shows trends in ASFRs using pooled data, and figure A3 displays the annual changes in ASFRs relative to the 1997 rates. It is evident from these figures that fertility declined at a relatively similar pace across all age groups, and fertility trends were not altered after 2003.

Figure A1 TFRs for women aged 15–39: Iraqi Kurdistan, 1997–2010

a. Data from separate 2006 and 2011 surveys

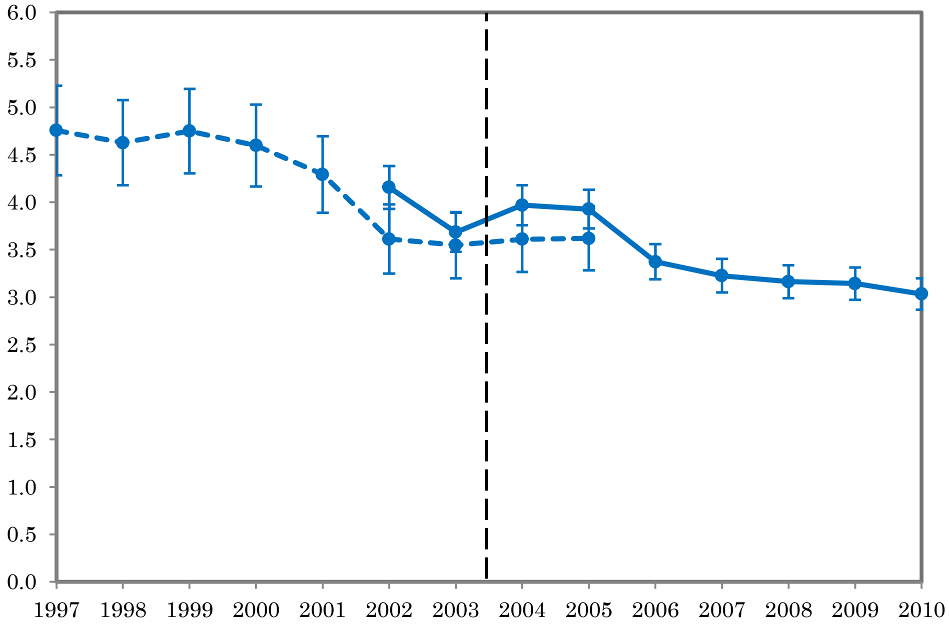

b. Pooled data

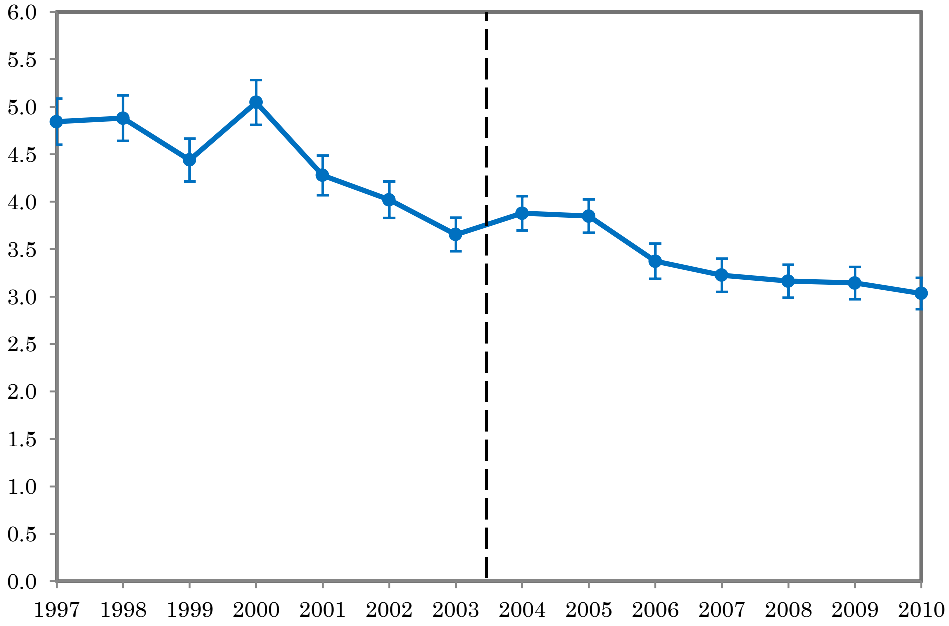

NOTE: The dashed and solid lines in top panel are fertility estimates from the 2006 and 2011 I-MICS, respectively.
SOURCE: 2006 and 2011 I-MICS.

Figure A2 ASFRs for women aged 15–39: Iraqi Kurdistan, 1997–2010

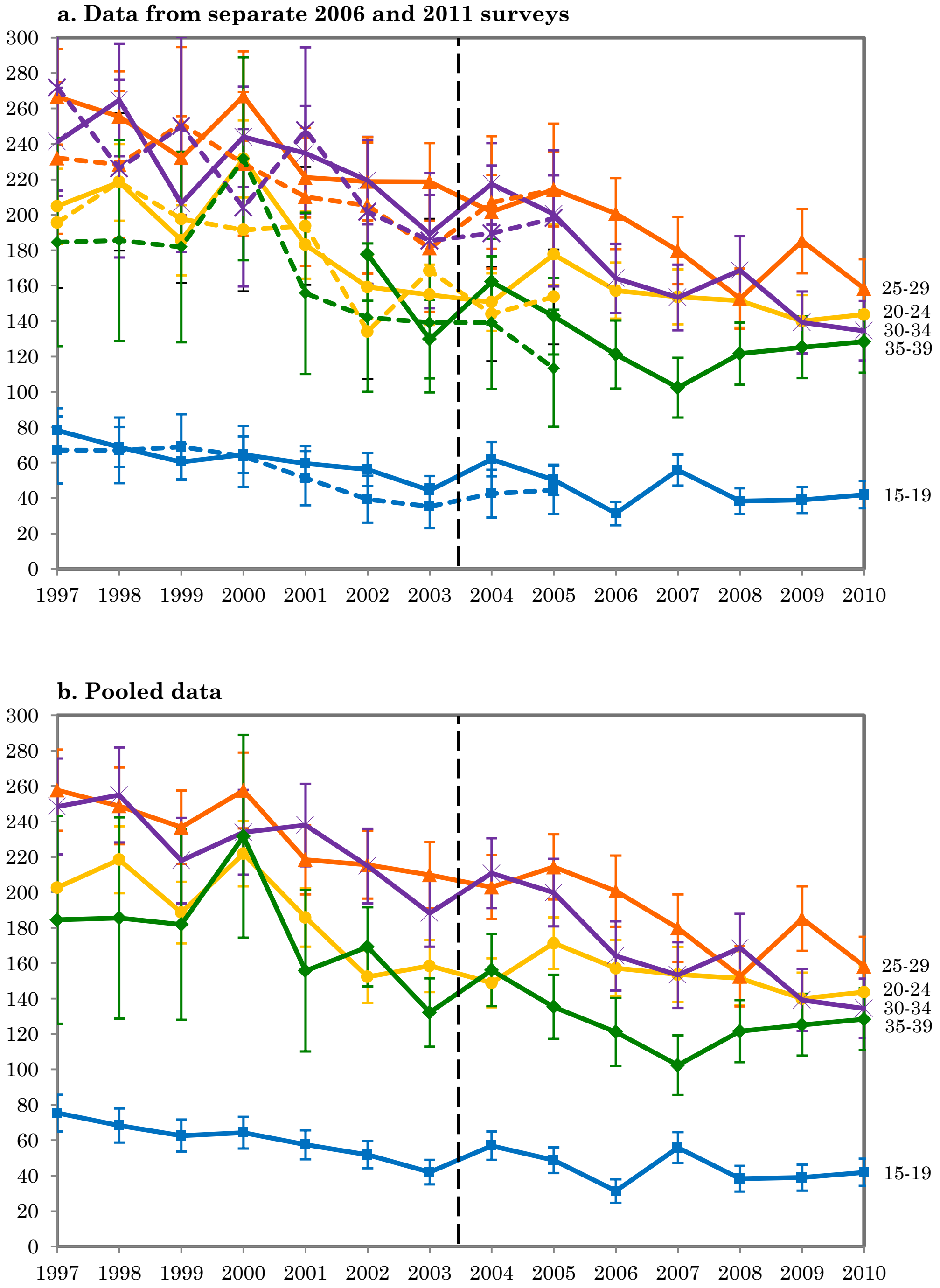

NOTE: The dashed and solid lines in top panel are fertility estimates from the 2006 and 2011 I-MICS, respectively.
SOURCE: 2006 and 2011 I-MICS.

Figure A3 ASFRs for women aged 15–39 relative to 1997 rates: Iraqi Kurdistan, 1997–2010 (Index: 1997 = 100)

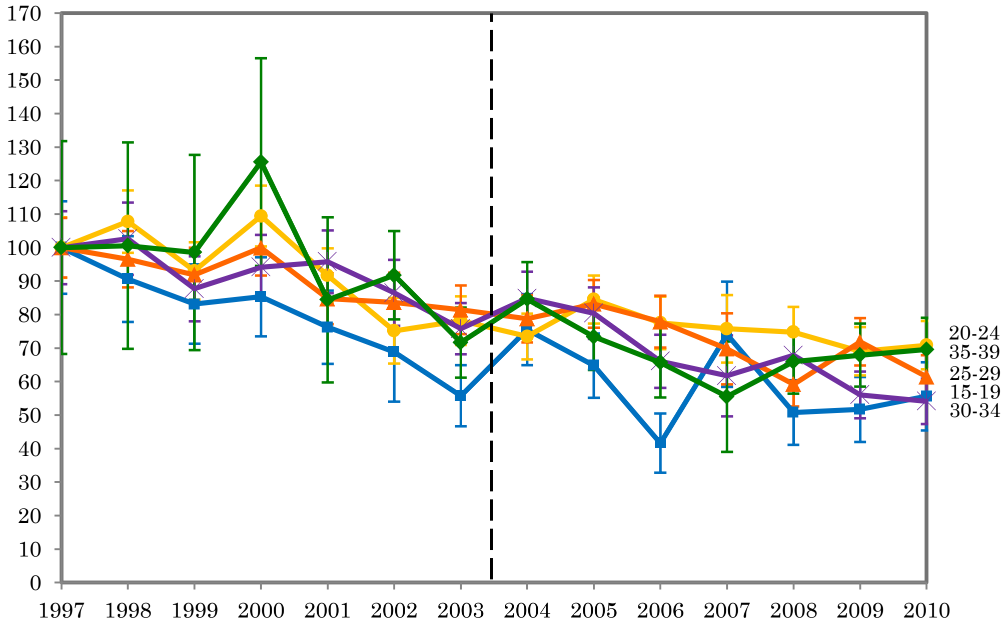

SOURCE: Pooled data from 2006 and 2011 I-MICS.
